# Improving Blood Flow Visualization of Recirculation Regions at Carotid Bulb in 4D Flow MRI Using Semi-Automatic Segmentation with ITK-SNAP

**DOI:** 10.3390/diagnostics11101890

**Published:** 2021-10-13

**Authors:** Minh Tri Ngo, Ui Yun Lee, Hojin Ha, Jinmu Jung, Dong Hwan Lee, Hyo Sung Kwak

**Affiliations:** 1 Department of Radiology of Hue Central Hospital, Hue, Thua Thien Hue 530000, Vietnam; ngominu@gmail.com; 2 Division of Mechanical Design Engineering, College of Engineering, Jeonbuk National University, Jeon-ju 54896, Korea; euiyun93@naver.com (U.Y.L.); jmjung@jbnu.ac.kr (J.J.); 3 Department of Mechanical and Biomedical Engineering, Kangwon National University, Chuncheon 24341, Korea; hojinha@kangwon.ac.kr; 4 Hemorheology Research Institute, Jeonbuk National University, Jeon-ju 54896, Korea; 5 Department of Radiology and Research Institute of Clinical Medicine of Jeonbuk National University, Biomedical Research Institute of Jeonbuk National University Hospital, Jeon-ju 54907, Korea

**Keywords:** recirculation regions, carotid bulb, four-dimensional flow magnetic resonance imaging, ITK-SNAP, computational fluid dynamics

## Abstract

Assessment of carotid bulb hemodynamics using four-dimensional (4D) flow magnetic resonance imaging (MRI) requires accurate segmentation of recirculation regions that is frequently hampered by limited resolution. This study aims to improve the accuracy of 4D flow MRI carotid bulb segmentation and subsequent recirculation regions analysis. Time-of-flight (TOF) MRI and 4D flow MRI were performed on bilateral carotid artery bifurcations in seven healthy volunteers. TOF-MRI data was segmented into 3D geometry for computational fluid dynamics (CFD) simulations. ITK-SNAP segmentation software was included in the workflow for the semi-automatic generation of 4D flow MRI angiographic data. This study compared the velocities calculated at the carotid bifurcations and the 3D blood flow visualization at the carotid bulbs obtained by 4D flow MRI and CFD. By applying ITK-SNAP segmentation software, an obvious improvement in the 4D flow MRI visualization of the recirculation regions was observed. The 4D flow MRI images of the recirculation flow characteristics of the carotid artery bulbs coincided with the CFD. A reasonable agreement was found in terms of velocity calculated at the carotid bifurcation between CFD and 4D flow MRI. However, the dispersion of velocity data points relative to the local errors of measurement in 4D flow MRI remains. Our proposed strategy showed the feasibility of improving recirculation regions segmentation and the potential for reliable blood flow visualization in 4D flow MRI. However, quantitative analysis of recirculation regions in 4D flow MRI with ITK-SNAP should be enhanced for use in clinical situations.

## 1. Introduction

Studies have shown that local hemodynamic conditions at the carotid bulbs, such as deceleration of blood flow or recirculation flow, have a significant impact on the initiation of atherosclerosis [1,2,3,4]. Therefore, assessment of blood flow through the carotid bulb is a topic of great interest in medical research [2,5,6] that could provide additional insight into vascular function in health and disease.

Four-dimensional (4D) flow magnetic resonance imaging (MRI) is based on phase-contrast (PC) MRI, which involves all fluid flow encoding resolved in three dimensions (3D) of space with time during the cardiac cycle [7]. Current 4D flow MRI acquisition schemes could provide information on the hemodynamics of human major arteries (aorta, pulmonary arteries, carotid arteries, and intracranial arteries) [8], but the trade-off between image resolution quality and scanning time still limits its widespread use in clinical practice [9].

One of the most important disadvantages of 4D flow MRI is a challenge in image segmentation caused by the low resolution and poor contrast between interested flow and surrounding tissues [10]. Additionally, complex flow in recirculation regions causes further segmentation problems in these 4D flow MRI datasets [11,12].

Manual blood vessel segmentation is a time-consuming process with low intra- and inter-operator repeatability and reproducibility. On the other hand, research has already focused on fully automatic methods. However, in many situations, fully automated segmentation is not possible or is not as effective as manual segmentation by experts with a thorough understanding of the underlying anatomy and pathology [13,14]. In this context, semi-automatic segmentation with subsequent manual improvement by vascular specialists could be a good solution, which could not only reduce the workflow time but also improve the segmentation accuracy for specific anatomical regions.

In this study, we improved the accuracy of 4D flow MRI carotid bulb segmentation, and, hence, subsequent recirculation regions analysis. ITK-SNAP segmentation software was included in the workflow for the semi-automatic generation of 4D flow MRI angiographic data. By using subject-specific computational fluid dynamics (CFD) as the ground truth model, we aimed to evaluate the feasibility of the proposed method in improving recirculation regions analysis of the human carotid bulb in 4D Flow MRI.

## 2. Materials and Methods

### 2.1. Subjects and Workflow

The subjects of this study were 7 healthy volunteers (age, 29.9 ± 0.4 years; 3 men and 4 women) who did not have a history of atherosclerotic plaques in either carotid artery, as confirmed by MRI. No risk factors such as hypertension, smoking, and diabetes were identified in the subjects. The bilateral carotid arteries from the 7 subjects (total = 14 carotid arteries) were examined. This study was approved by the research ethics committee of Jeonbuk National University Hospital and Jeonbuk National University Medical School (JUH2017-10-007), and signed informed consent was received from all the participating subjects.

**Figure 1:**
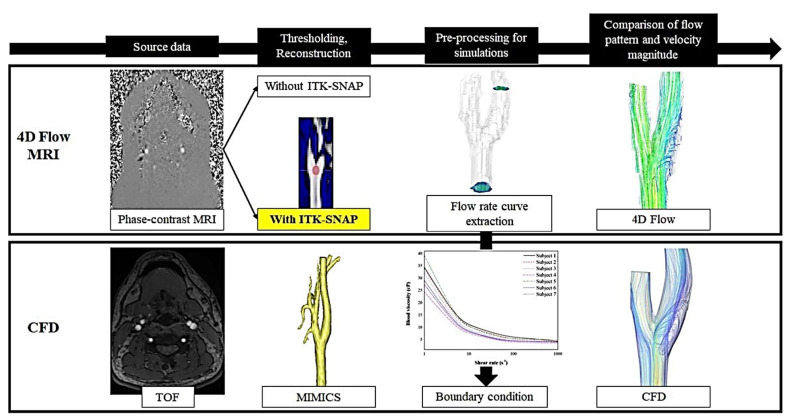
Flow chart of the hemodynamic analysis process of 4D flow MRI (**first row**) and CFD (**second row**). Segmentation using ITK-SNAP was included in the 4D flow MRI workflow.

The study flow chart is illustrated in Figure 1. For the 4D flow MRI workflow, the ITK-SNAP (ITK-SNAP 3.8.0, http://itksnap.org accessed on March of 2021) method [15] was utilized to improve carotid bulb region segmentation before 4D flow MRI analysis using the source data from PC MRI. For the CFD simulations, time-of-flight (TOF) MRI source data was used for re-construction of the carotid artery by the MIMICS program. The flow rate curves of the common carotid artery (CCA) and internal carotid artery (ICA) were extracted from the 4D flow MRI analysis to apply a boundary condition on CFD simulations. The blood flow visualization and velocity magnitudes were compared between 4D flow MRI analysis and CFD simulations.

### 2.2. MRI Scan

A 3-T MRI system (Magnetom Skyra, Siemens, Erlangen, Germany) was used to per-form TOF MRI sequences. The used parameters were as follows: slice thickness: 0.8 mm, repetition time: 24, echo time: 3.35, number of averages: 1, flip angle: 15°, field of view: 131 × 219 mm, resolution: 3.5 pixels per mm, and voxel size: 0.3 × 0.3 × 0.8 mm^3^.

All source data for 4D flow MRI analysis was acquired based on PC MRI with 3D velocity encoding. The following parameters were set for the PC MRI: velocity encoding: 100 cm/s, cardiac number of images: 12 to 22, slice thickness: 1.1 mm, repetition time: 47.4, echo time: 3.16, number of averages: 1, flip angle: 20°, field of view: 194 × 194 mm, resolution: 0.9 pixels per mm, and voxel size: 1.1 × 1.1 × 0.07 mm^3^.

### 2.3. MRI Data Segmentation and Processing

The 4D flow MRI source data is entered in a software package (MATLAB, Natick, MA, USA) for background correction and phase unwrapping [1,16]. The image segmentation is then performed by using the ITK-SNAP software to create masking in-phase images to differentiate the fluid area from the external static tissues. To create the mask images from the angiograms, semi-automatic segmentation was generated based on pre-knowledge of the vessel anatomy of interest followed by manual revising of the resulting segmentation using a specific tool of ITK-SNAP [15]. In the level-set segmentation, we set a seed point for the segmentation and used gradient mapping to drive the segmentation’s evolution [17,18]. Seed points were placed in the carotid bifurcation for every subject (Figure 1). The arterial segmentation proceeded until the ICA, external carotid artery (ECA), and CCA were fully captured. Since the current framework was crucial in the present study, 2 experienced radiologists double-checked all the segmentation processes.

By integrating the ITK-SNAP segmentation software and conventional post-processing tools (Ensight, CEI, Apex, NC, USA), 4D flow MRI analysis was conducted allowing interactive quantification and visualization of the vessel of interest.

### 2.4. 3D Reconstruction of Carotid Artery for CFD

According to the previous study, various segmentation techniques have been proposed including an algorithm for automated segmentation of blood vessels and for generating a centerline [19]. For the reconstruction of carotid artery geometry from 2D to 3D, the output of TOF MRI source data was imported to the MIMICS software package (version 20.0; Materialise NV, Leuven, Belgium). The thresholding method was applied considering the 3D-converted blood vessels not to be distorted. Two experienced radiologists (more than 10 years experience) confirmed and verified the segmentation process. The crop method was performed to designate the carotid artery. Unnecessary branches at the ECA were eliminated using the 3D edit mask module. The reconstructed carotid artery was saved as a standard tessellation language (STL) file for CFD simulations.

### 2.5. Computational Fluid Dynamics

**Figure 2:**
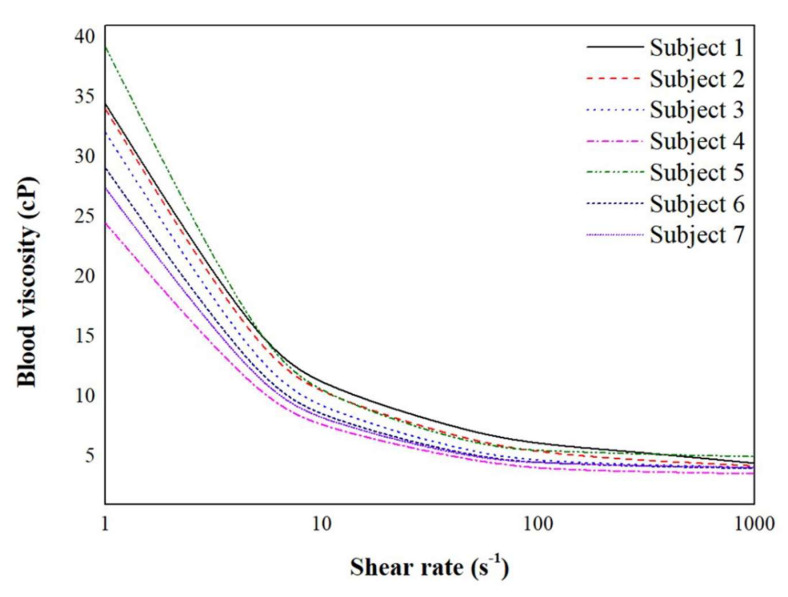
The whole blood viscosity profiles for each subject according to shear rate. Subject-specific blood viscosity profiles are shown with different lines.

The saved STL format of geometry was imported to the COMSOL Multiphysics 5.2 a software package (COMSOL Inc., Burlington, MA, USA) for CFD simulations. The continuity and Navier-Stokes equations were set as convergence criteria. Iterative solver (generalized minimal residual algorithm (GMRES)) was set for the numerical simulation. For the convergence of the solution, the value of 0.01 of relative tolerance was applied. Blood flow was set as an incompressible non-Newtonian fluid. The subject-specific blood viscosity was measured using a scanning capillary tube viscometer (Rheovis-01; Biorheologics Co., Ltd., Jeonju, South Korea). The blood viscosity profiles for each subject are shown in Figure 2. Based on the Casson model, the non-Newtonian blood viscosity profiles according to the shear rate were obtained. The measured subject-specific Casson constant and yield stress were used for parameters of the CFD simulations.

(1) $$\sqrt{\tau \;}=\sqrt{\tau_{y}}+\sqrt{k}\sqrt{\dot{\gamma }}\;\mathrm{when}\;\tau >{\;\tau }_{y}\;\dot{\gamma }=0\;\mathrm{when}\;\tau <{\;\tau }_{y}$$

In the Casson model (Equation (1)), where τ is wall shear stress, $\tau_{y}$ is the yield stress. k denotes a Casson constant, and $\dot{\gamma }$ indicates the shear rate. The vessel wall was assumed as rigid with a no-slip flow. For inlet (CCA) and outlet (ICA) boundary conditions, the extracted subject-specific flow rate from flow MRI and inlet area were used to obtain velocity profiles during the cardiac cycle. A traction-free boundary condition was applied at the ECA. To avoid transient numeric errors, three cardiac cycles were solved, and the second cycle was utilized for quantification and visualization.

### 2.6. Comparison of Visualization and Quantification

**Figure 3:**
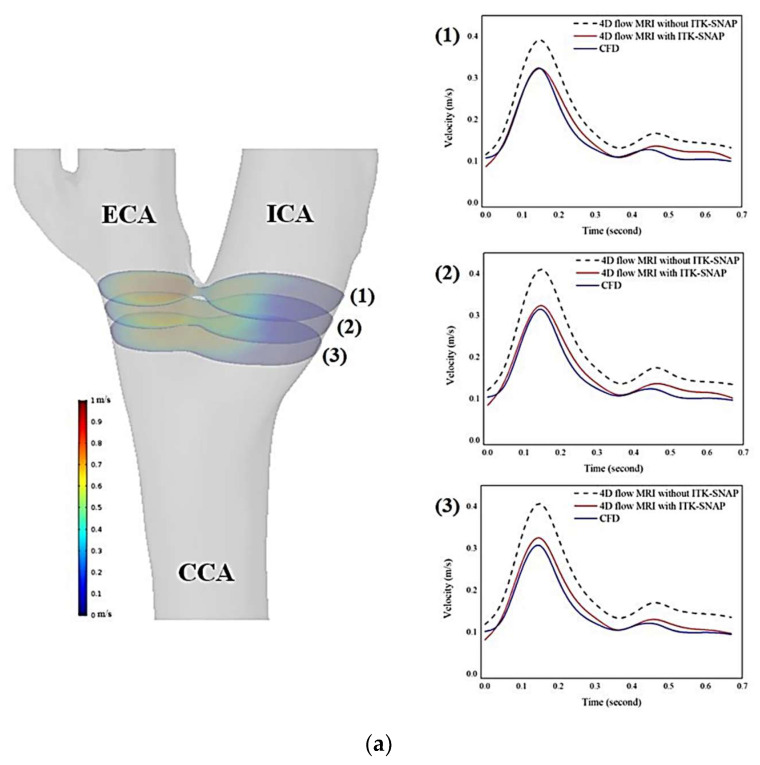

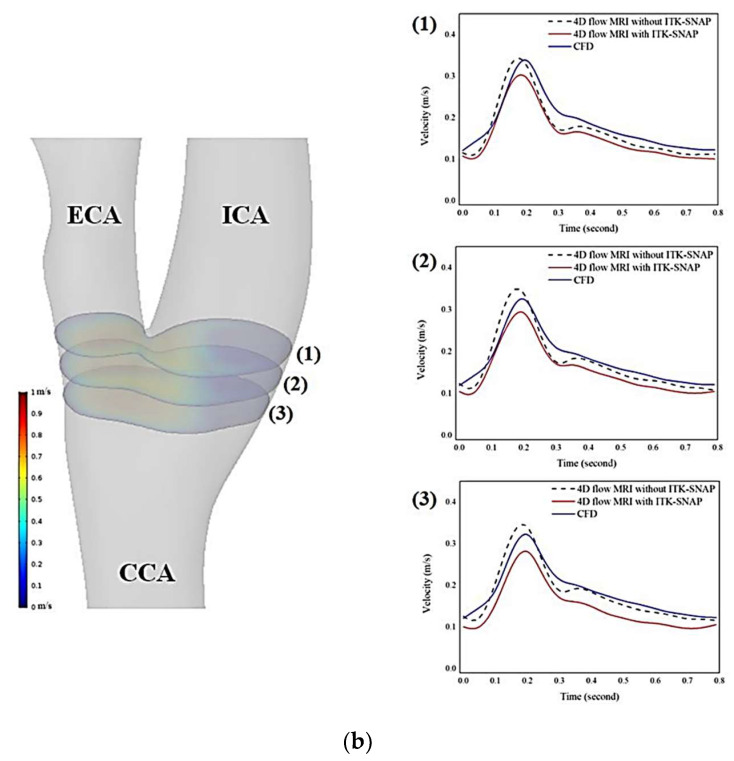
(**a**) Three analysis planes of left carotid artery from subject 1 (**left panel**). The velocity waveforms at the bifurcation planes provided by 4D flow MRI without ITK-SNAP (dashed line), 4D flow MRI with ITK-SNAP (red line), and CFD (blue line). Results from 4D flow MRI with ITK-SNAP and CFD revealed a close similarity of shape. (**b**) Three analysis planes of left carotid from subject 5 (**left panel**). The velocity waveforms at the bifurcation planes provided by 4D flow MRI without ITK-SNAP (dashed line), 4D flow MRI with ITK-SNAP (red line), and CFD (blue line). The velocity waveforms were variable among the 3 approaches.

Three cross-sectional planes were positioned at the carotid bulb, as shown in Figure 3. The interval distance of each plane was 1.5 mm. The velocity profiles for each plane were considered to compare between 4D flow MRI analysis with and without ITK-SNAP and CFD simulations. To evaluate the improvement of blood flow visualization at recirculation regions, the velocity with blood flow streamlines at peak-systole were used. Moreover, 3D vector arrows were used to compare flow directions at the recirculation regions.

### 2.7. Statistical Analysis

Using the Bland and Altman approach, the calculated mean blood flow velocities were assessed in all 42 analysis planes for all time frames during one cardiac cycle (95% limits of agreement). The analysis was conducted using the statistical program package SPSS, version 21.0 (SPSS Inc., Chicago, IL, USA).

## 3. Results

### 3.1. Correlation of Velocity Measurement between 4D Flow MRI and CFD

For comparison between 4D flow MRI and CFD simulation, the blood flow velocity was calculated at 3 cross-sectional planes. Two subjects were analyzed as representative cases. The left carotid arteries of subject 1 and subject 5 are shown in Figure 3a,b, respectively. The velocity graphs for each cross-sectional plane are shown on the right side of the carotid artery model. Each graph contains the velocity values according to time, and the velocity values were analyzed with three different approaches (4D flow MRI without ITK-SNAP, 4D flow MRI with ITK-SNAP, and CFD simulation). In Figure 3a, 4D flow MRI without ITK-SNAP was 30.20% overestimated compared to CFD simulation at peak-systole (cross-sectional plane (3)). 4D flow MRI with ITK-SNAP showed a 4.69% quantitative difference compared with CFD analysis at peak-systole (cross-sectional plane (3)).

Unlike with subject 1, subject 5 showed a greater difference between 4D flow MRI with ITK-SNAP and CFD than the difference between 4D flow MRI without ITK-SNAP and CFD as indicated in Figure 3b. At peak-systole, 4D flow MRI without ITK-SNAP was 2.44% overestimated compared to CFD simulation (cross-sectional plane (2)). Further, 4D flow MRI with ITK-SNAP was 10.07% underestimated compared with CFD analysis.

**Figure 4:**
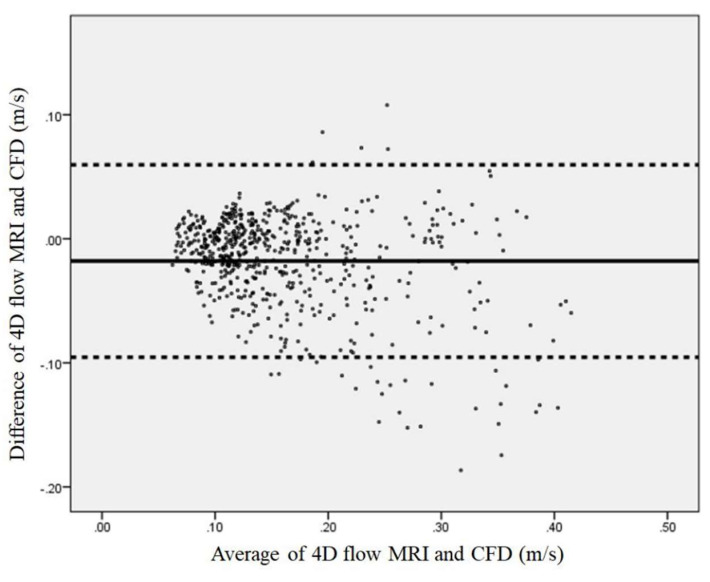
Bland–Altman plot analysis of 14 carotid bifurcation velocity data (*n* = 606) comparing results of mean blood flow velocity over the cardiac cycle for all 4D flow MRI with ITK-SNAP and CFD analysis planes. The solid line indicates the mean of the difference between 4D flow MRI and CFD. The dashed lines represent the limits of agreement between the two methods, from −1.96 to +1.96 of standard deviation (limit range from −0.095 to 0.060 m/s).

Bland–Altman plot analysis of velocity data of 14 carotid arteries (*n* = 606) comparing the results of mean blood flow velocity over the cardiac cycle for all analysis planes obtained from 4D flow MRI with ITK-SNAP and CFD is shown in Figure 4. Although the dispersion relative to the local measurement errors between the two methods still existed, most of the data points were inside of the 95 percent limit band (limit range from −0.095 to 0.060 m/s), indicating reasonably good agreement between velocity measurements of 4D flow MRI with ITK-SNAP and CFD.

### 3.2. Impact on Blood Flow Visualization at Recirculation Regions

**Figure 5:**
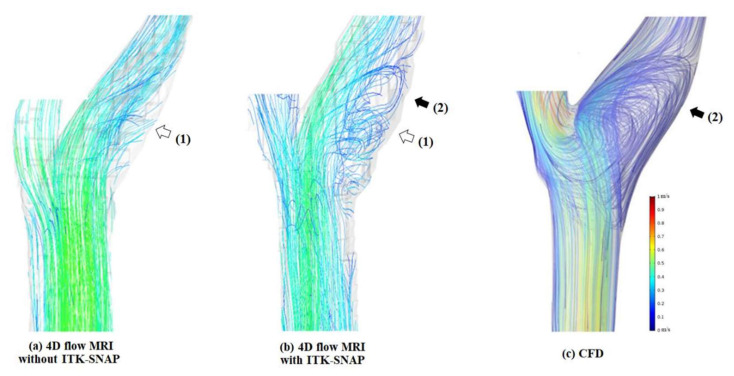
The 3D blood flow characteristics at carotid bulb using 3 different approaches. (**a**) 4D flow MRI without ITK-SNAP) and (**b**) 4D flow MRI with ITK-SNAP: the visualization of recirculation blood flow at carotid bulb is clearly improved by using ITK-SNAP (white arrow labeled 1); (**b**) and (**c**) computational fluid dynamics: CFD): the major flow and secondary flow structures observed by 4D flow MRI with ITK-SNAP and CFD coincide (black arrow labeled 2).

As seen in Figure 5, 3D blood flow characteristics of a representative subject were displayed by using three different approaches. By comparing the blood flow visualization at the carotid bulb before and after segmentation with the proposed method, the streamline length of recirculation regions was greatly improved after the correction as pointed with the white arrow (1). The recirculation region was found in both 4D flow MRI with ITK-SNAP and CFD as indicated with the black arrow (2).

**Figure 6:**
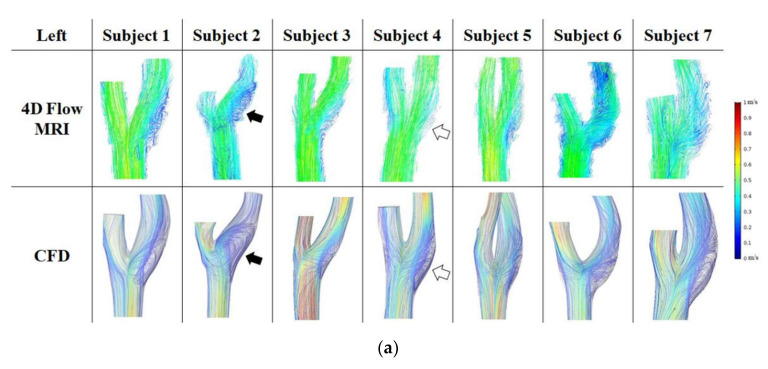

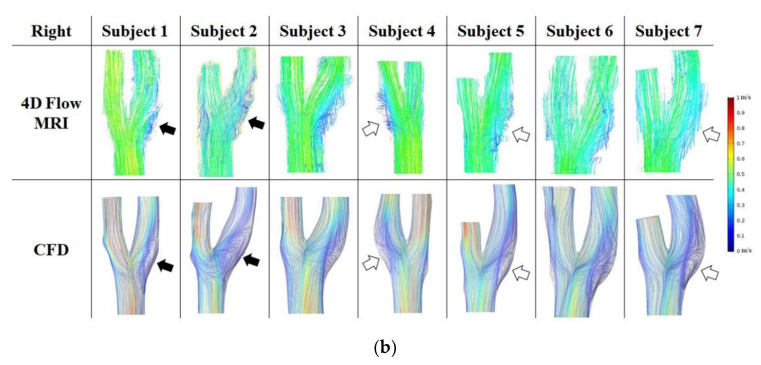
(**a**) The 3D flow patterns for seven left carotid arteries at peak systole observed by 4D flow MRI with ITK-SNAP (**first row**) and CFD (**second row**). (**b**) The 3D flow patterns for seven right carotid arteries at peak systole observed by 4D flow MRI with ITK-SNAP (**first row**) and CFD (**second row**).

The comparison of blood flow streamlines between 4D flow MRI with ITK-SNAP group and CFD are displayed in Figure 6, and Figure 6a,b show the left carotids and right carotids, respectively. The black arrow indicates similarity, and the white arrow represents difference. In Figure 6a, subject 2 had an improved recirculation region on the carotid bulb as in the CFD simulation. However, subject 4, showed a great difference between the two methods. An error was found in that the blood flow was not connected in the carotid bulb.

On the right carotid arteries, various blood flow differences occurred between the two groups (Figure 6b). In 4D flow MRI with ITK-SNAP, the blood flow seemed to be cut and disappeared in the recirculation region due to low resolution (subject 4 and 5), or the blood flow went straight in the carotid bulb (subject 7).

**Figure 7:**
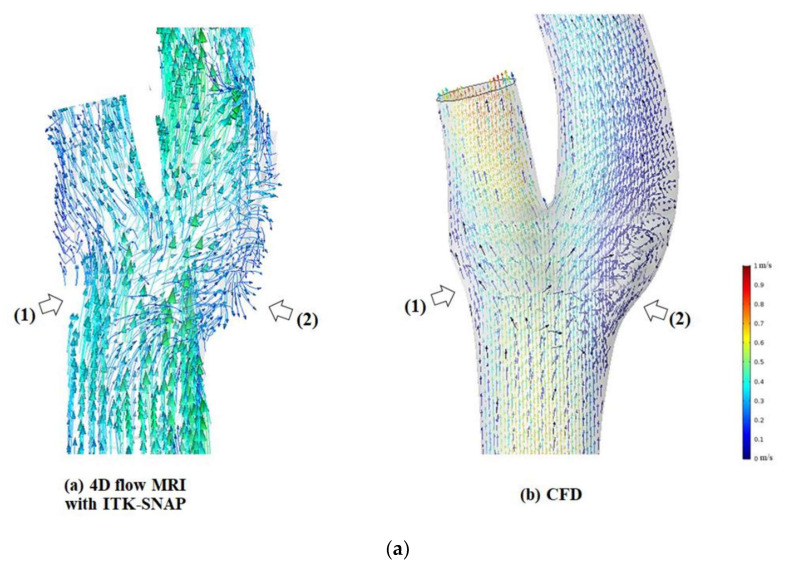

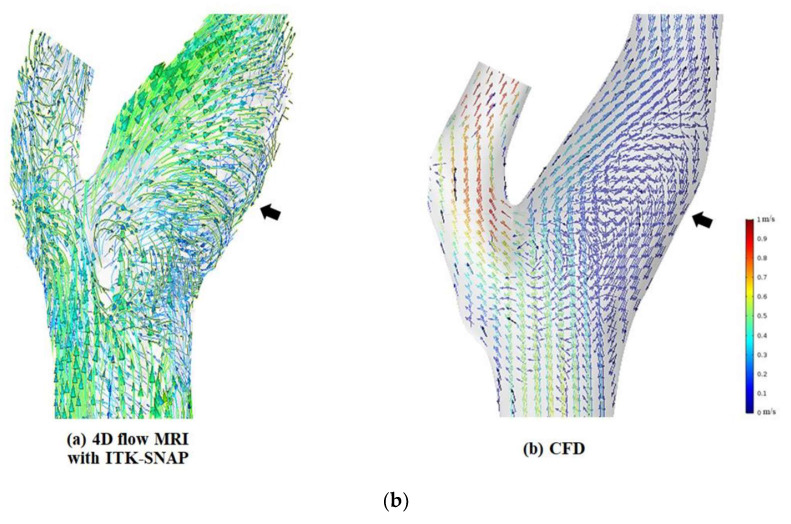
(**a**) The 3D vector fields derived from 4D flow MRI with ITK-SNAP (**left**) and CFD (**right**). Several streamlines were disconnected at the segment of an arterial wall within the carotid bifurcation domain in 4D flow MRI (white arrows labeled 1). Disagreement in secondary flow directions at the peripheral area of recirculation regions between the two techniques (white arrows labeled 2). (**b**) The 3D vector fields derived from 4D flow MRI with ITK-SNAP (**left**) and CFD (**right**). Generally, 4D flow MR images of secondary flow directions (black arrows) have similarities with CFD simulation.

Flow visualization with a vector is shown in Figure 7. Several streamlines suddenly stopped at a section of the arterial wall within the carotid bifurcation area in 4D flow MRI (Figure 7a at point (1)). In several simulations, disagreement was found in secondary flow directions at the peripheral area of recirculation regions between the two techniques (Figure 7a at point (2)).

Despite these deviations, in general, visual comparison between the two techniques shows that the major flow and the recirculation flow structures were observed in 4D flow MRI at the carotid artery bifurcation domain, which was similar to the results of CFD simulation (Figure 7b). The 3D vector fields derived from the proposed methods of 4D flow MRI and CFD showed good agreement in the directions of recirculation flow at the carotid bulbs.

## 4. Discussion

We conducted a method to semi-automatically reconstruct 3D segmentation of recirculation regions at the carotid bulb from 4D flow MRI data by using ITK-SNAP software. Compared with subject-specific CFD simulation results, the velocity measured by 4D flow MRI without ITK-SNAP was higher (Figure 3a). This discrepancy can be attributed to the fact that the standard 4D flow MRI analysis was derived from thresholding of the vessel, and the recirculation regions were almost ignored due to the limited resolution of PC MRI [9,12,20]. As a result, a subset of these significant voxels with important velocity information of the recirculation area was most likely missed. Typically, this limitation is predominantly present in the vessel bifurcation, where the vessels are tortuous and hence cannot be filled by as many voxels. The present workflow handles these issues by performing a semi-automated segmentation procedure with ITK-SNAP. Consequently, we manually revised the specific components of the vessel segmentation. Our resulting segmentations are almost certainly of higher accuracy than what is performed by standard 4D flow MRI methods.

However, similar to previous studies [21,22,23,24], our results of 4D flow MRI with ITK-SNAP analysis still tend to measure lower flow velocities than CFD in several subjects (Figure 3b). This is due to the deviation in the geometric angiographic reconstruction between the two methods. While 4D flow MRI analysis is based on PC MRI, CFD simulations applied complementary vessel geometry information from TOF-MRI, which have higher contrast and resolution than PC MRI. 4D flow MRI geometry is assumed as a maximum intensity projection of 3D space with all timeframes [11]. As a result of the 4D flow MRI segmentation of anatomical models, a greater vessel caliber than CFD was estimated, resulting in a lower blood flow velocity in 4D flow MRI measurements compared to CFD simulations [25].

As shown in Figure 4, despite the relatively good agreement in terms of velocity calculated at the carotid bifurcation between 4D flow MRI with ITK-SNAP and CFD, the dispersion of velocity data points associated with deviations of measurement between the two techniques exists. Most of the average differences between the measurements lie under the lower limit band (−1.96 of standard deviation). The inferior performance of the proposed approach is associated with the velocity encoding (VENC) level setting of the scan parameter. The VENC level is frequently set high enough to prevent aliasing in the regions with the highest velocity. However, this resulted in non-optimal results for recirculation regions, which still showed a high velocity-to-noise ratio [26,27]. Methods to use multiple point encoding or multiple VENCs would help to improve the conditions of low-flow at recirculation areas [27,28].

Another popular technique is the creation of angiographic images from phase-contrast MRI, which focuses especially on major flow. However, slower blood flows frequently present in reality, such as in recirculation regions at the carotid bulb, and are typically not clearly visible in PC MRI [11,12]. Furthermore, one of the main drawbacks of 4D flow MRI is the lack of an obvious distinction between the blood flow and the tissues that it passes through, which further exacerbates the segmentation problem in these datasets [29]. When comparing previous 4D flow MRI results with the new framework by interoperating with ITK-SNAP segmentation, our proposed approach demonstrated efficacy in improving recirculation regions segmentation quality in 4D flow MRI, and it has the potential for reliable blood flow visualization. Moreover, while the present study used a free software package (ITK-SNAP [14]), the proposed technique can be readily and widely applied in this field.

In 3D blood flow visualization, our study still identified several differences between 4D flow MRI with ITK-SNAP and CFD. The influence of a partial volume effect was still observed from the shape of 4D flow MRI profiles [20]. It is therefore still challenging to map such behaviors with an appropriate resolution in conventional 4D flow MRI [9]. However, despite a lower resolution in the observation of recirculation regions, our proposed 4D flow MRI approach of recirculation flow directions has similarities with CFD simulation. Both techniques represent a consensus in blood flow characteristics at the carotid bulb where flow recirculation occurs. This could provide additional insight into the physiology of carotid artery bifurcation blood flow and prediction of high-risk atherosclerotic plaque initiation at the carotid bulbs [30].

Our study had several limitations. The small number of study participants is one of its limitations. In addition, we are still limited in part of the workflow with a manual segmentation process. Despite taking great care when conducting angiographic segmentation, it was still difficult to consistently segment the carotid bulb while excluding with high specificity the jugular veins and the small ECA branches. However, we do not intend to rely on manually revised segmentation for our work, but we believe our resulting segmentation will be comparable to those obtained using more advanced techniques that could be established in the future. Moreover, the use of a 4D flow MRI algorithm as a boundary condition for CFD simulation creates a bias in the subsequent comparison of these two approaches. However, the use of analysis planes at the carotid bifurcation, which has a distance with the outlet and inlet boundary conditions, can aid in reducing this bias. A further limitation involves the spatial resolutions used in 4D flow MRI scans. In a conventional 4D flow MRI, the resolution could not be greater than 0.9 pixels per mm with a scan time of 20 min. Another drawback is the lack of assessment of the measurements’ intra- and inter-operator repeatability and reproducibility. Finally, while the agreement between 4D flow MRI with ITK-SNAP and CFD for blood flow velocity measurement is useful in healthy subjects, further study is needed to decide whether it is also accurate for use in carotid disease.

## 5. Conclusions

Assessment of recirculation regions using 4D flow MRI requires precise segmentations of the underlying anatomy. This study proposes an approach for improving carotid bulb analysis in 4D flow MRI data by incorporating it with ITK-SNAP segmentation software. Comparing 4D flow MRI analysis and subject-specific CFD simulations, our proposed strategy showed the feasibility of improving recirculation regions segmentation and the potential for reliable blood flow visualization in 4D flow MRI. However, quantitative analysis of recirculation regions in 4D flow MRI with ITK-SNAP should be enhanced for use in clinical situations.

## Data Availability

The data presented in this study are available upon request from the corresponding author. The data are not publicly available due to privacy restrictions.
